# Comment on “intra-articular hip injections for lateral hip pain” by M. C. Bessette *et al*.

**DOI:** 10.1093/jhps/hnv003

**Published:** 2015-01-29

**Authors:** Jorm M. Nellensteijn

**Affiliations:** Spire Cambridge Lea Hospital, Department of Orthopedics, Cambridge, UK

With interest I was reading the article about intra-articular hip injections for lateral hip pain by Bessette *et al*. [1].

Refractory greater trochanteric pain syndrome (GTPS) is a common and challenging problem in daily practice. It is interesting to see that intra-articular injection might be a solution to a seemingly extra-articular problem. A few comments and questions rose to my mind.

The authors state the diagnosis of GTPS was made clinically, although some patients had magnetic resonance imaging (MRI) available at the time of initial consultation. In Table I, all patients except two have MRI outcomes. Most of the MRI scans show intra-articular pathology. This suggests that at time of inclusion, the MRI outcomes were known and could be a form of bias.

Inclusion criteria required patients to have lateral hip pain for at least 6 months, at least one prior trochanteric corticosteroid injection, one course of NSAID and physiotherapy (PT). Unfortunately, no further comment is made about the type of PT patients had followed. After inclusion, directed PT by a therapist familiar with hip-specific exercises, were given.

We know that an intra-articular hip block is a perfect differentiating tool in hip-spine dilemma. I am not sure if it is as helpful to differentiate intra-articular from extra-articular pathologies. The extra-articular structures are close to the joint capsule, and we have to assume that the capsule is watertight and no leakage or spreading will occur.

Patients experienced significant improvements at 1 and 12 weeks. It is interesting to know the result from the Marcaine after a few hours, as usually is recorded after a hip joint block. Sadly this is not mentioned.

Could it be possible that the 1-week improvement is due to the anaesthetic and 12-week improvement because of a functional hip protocol?

In short, are we looking at the result of injection or at a well-established PT program?

Unfortunately, only 9 of the 16 (56%) patients were reviewed after 1 week and only 7 (44%) patients after 12 weeks. The authors state that a larger cohort with better adherence to protocol would make the results more robust. I think that it might not make the results more robust but could show us a total different outcome.

Because of the aforementioned issues, small study size and the variable follow-up, one should be very careful to drawing conclusions from this study.

I agree with the authors’ conclusion this study leaves room for future research into this matter.
